# Ocean model resolution dependence of Caribbean sea-level projections

**DOI:** 10.1038/s41598-020-71563-0

**Published:** 2020-09-03

**Authors:** René M. van Westen, Henk A. Dijkstra, Carine G. van der Boog, Caroline A. Katsman, Rebecca K. James, Tjeerd J. Bouma, Olga Kleptsova, Roland Klees, Riccardo E. M. Riva, D. Cornelis Slobbe, Marcel Zijlema, Julie D. Pietrzak

**Affiliations:** 1 Institute for Marine and Atmospheric Research, Utrecht University, Princetonplein 5, 3584 CC Utrecht, The Netherlands; 2 Department of Hydraulic Engineering, Civil Engineering and Geosciences, Delft University of Technology, Stevinweg 1, 2628 CN Delft, The Netherlands; 3 Estuarine and Delta Systems, Royal Netherlands Institute for Sea Research and Utrecht University, Korringaweg 7, 4401 NT Yerseke, The Netherlands; 4 Department of Geoscience and Remote Sensing, Civil Engineering and Geosciences NL, Delft University of Technology, Stevinweg 1, 2628 CN Delft, The Netherlands

**Keywords:** Ocean sciences, Physical oceanography, Ocean sciences, Physical oceanography, Climate sciences, Climate change, Climate and Earth system modelling, Projection and prediction

## Abstract

Sea-level rise poses severe threats to coastal and low-lying regions around the world, by exacerbating coastal erosion and flooding. Adequate sea-level projections over the next decades are important for both decision making and for the development of successful adaptation strategies in these coastal and low-lying regions to climate change. Ocean components of climate models used in the most recent sea-level projections do not explicitly resolve ocean mesoscale processes. Only a few effects of these mesoscale processes are represented in these models, which leads to errors in the simulated properties of the ocean circulation that affect sea-level projections. Using the Caribbean Sea as an example region, we demonstrate a strong dependence of future sea-level change on ocean model resolution in simulations with a global climate model. The results indicate that, at least for the Caribbean Sea, adequate regional projections of sea-level change can only be obtained with ocean models which capture mesoscale processes.

## Introduction

The ongoing increase of global sea level threatens both coastal and low-lying regions. The combination of future sea-level rise with increasing storm occurrence and intensity may exacerbate beach erosion^1^. This can have severe consequences for areas which are highly dependent on their beaches, either for flood safety or economically for local tourism^2,3^. Higher sea levels can also lead to changes in coastal ecosystems, and permanent submergence of land and human settlements^4^. For making adequate decisions on the development of successful adaptation strategies to sea-level rise, skillful projections over the next decades are crucially important^5^.

By decomposing the components contributing to the satellite-observed global mean sea-level (GMSL) rise between 1993 and 2014, it was shown that the dominant contributor to GMSL is thermal expansion of the ocean^6^. Over the same period, the contribution of mass loss of both glaciers and large ice sheets to GMSL rise became more important over time^6,7^. On a regional scale, sea-level rise may deviate from GMSL rise^8^ and can be caused by other processes than thermal expansion. For example, the dominant contributor to sea-level rise in the Caribbean is thermal expansion ($40\% = 0.8\,\hbox {mm}\,\hbox {year}^{-1}$) while east of the Caribbean^9^ this is ocean mass redistribution ($50\% = 1.7\,\hbox {mm}\,\hbox {year}^{-1}$). Regional sea-level change induced by variations in the gravitational contribution due to large ice sheets and glacial isostatic adjustment is very homogeneous over these two regions^9^. Hence, the difference in magnitude of sea-level rise in the different regions is caused by ocean sterodynamic effects (i.e. mass redistribution and thermal expansion)^10^.

Ocean volume conserving climate models, such as those used in the sixth coupled model inter-comparison projects (CMIP) of the Intergovernmental Panel on Climate Change (IPCC) Assessment Report, provide projections of the dynamic sea level (DSL) and sterodynamic sea level (SDSL)^22^. Due to their low spatial resolution, the ocean component of the CMIP6 models (typically $1^{\circ }$ horizontal resolution) only includes a parameterisation of the effects of mesoscale processes on transport properties, e.g., of heat and salt^11^. One way to overcome the limited ocean resolution is by applying dynamical downscaling techniques in a regional domain^12–14^ which is forced by a coarser global climate model. However, this technique also has its limitations as sea-level variability in such a global climate model is resolution dependent^15,16^. From globally eddy-resolving ocean model simulations^17,18^, it is known that explicitly capturing eddies leads to substantially different DSL responses compared to lower-resolution models, for example through the modification of boundary currents. Regional sea-level projections based on global climate models which parameterise mesoscale processes (such as in CMIP6) therefore miss relevant physics^19^ affecting the projected sea-level rise at the end of the century.

To study the effect of ocean model resolution on DSL and SDSL projections, we here analyse the Caribbean region. This is a typical region where large effects can be expected as it lies downstream of a strong western boundary current, the North Brazil Current (NBC). This current is characterised by the shedding of mesoscale ocean eddies which strongly affect the downstream region, the Caribbean Sea. The NBC is also part of the larger Atlantic Meridional Overturning Circulation (AMOC) which is expected to weaken under climate change^20,21^. Both the large-scale ocean circulation and the mesoscale eddies are expected to be much better represented in a high-resolution ocean model compared to a low-resolution one and here below we explore the consequences for regional (stero)dynamic sea-level projections.

## Climate model simulations

In order to systematically investigate the effect of ocean model resolution on simulated Caribbean sea-level rise, we performed simulations with the Community Earth System Model (CESM). The CESM is a fully-coupled climate model with a volume conservation constraint for the ocean component. The CESM has no dynamic ice sheet model and hence the effects of mass loss by glaciers and of the Greenland- and Antarctic ice sheets are not captured. The high-resolution version of CESM has an ocean component with a 10 km ($0.1^{\circ }$) horizontal resolution capable of capturing the development and interaction of mesoscale ocean eddies^11^ and an atmosphere component with a horizontal resolution of 50 km ($0.5^{\circ }$). The ocean (atmosphere) component of the low-resolution version of the CESM has a horizontal resolution of 100 km (125 km). The ocean component of this low-resolution model does not capture mesoscale processes.

With the high-resolution version, we first performed a 200 years spin-up with seasonally varying yearly-repeated forcing conditions (aerosols, solar insolation) of the year 2000 (with a $$\hbox {pCO}_2$$ level of 369 ppmv). After the 200 year spin-up period, we branched two simulations, one in which the spin-up was further extended (HR-CESM Control) and one in which the atmospheric $$\hbox {pCO}_2$$ increases by about $$1\%$$ each year (HR-CESM) for 101 years (Supplementary Figure S1). The HR-CESM simulation is the first of its kind due to its extremely high computational costs. The low-resolution version of the CESM was spun-up with a present-day configuration (similar to the HR-CESM) for 1,200 years and we branched a control simulation (LR-CESM Control) and a simulation with about $$1\%$$$$\hbox {pCO}_2$$ increase each year (LR-CESM) for 101 years. Both the control simulations are almost free of any temperature trends over the upper 1,000 m of the ocean compared to the HR-CESM and LR-CESM simulations (Supplementary Figure S2). More details of the CESM simulations are provided in “Methods” section.

The majority of the CESM output is stored as monthly-averaged quantities. These monthly-averaged quantities are converted to yearly averages which are used in the analyses below. A limited number of quantities are stored as daily averages which are used in generalised extreme value analysis (see “Methods”).

## Sterodynamic sea-level trends

**Figure 1:**
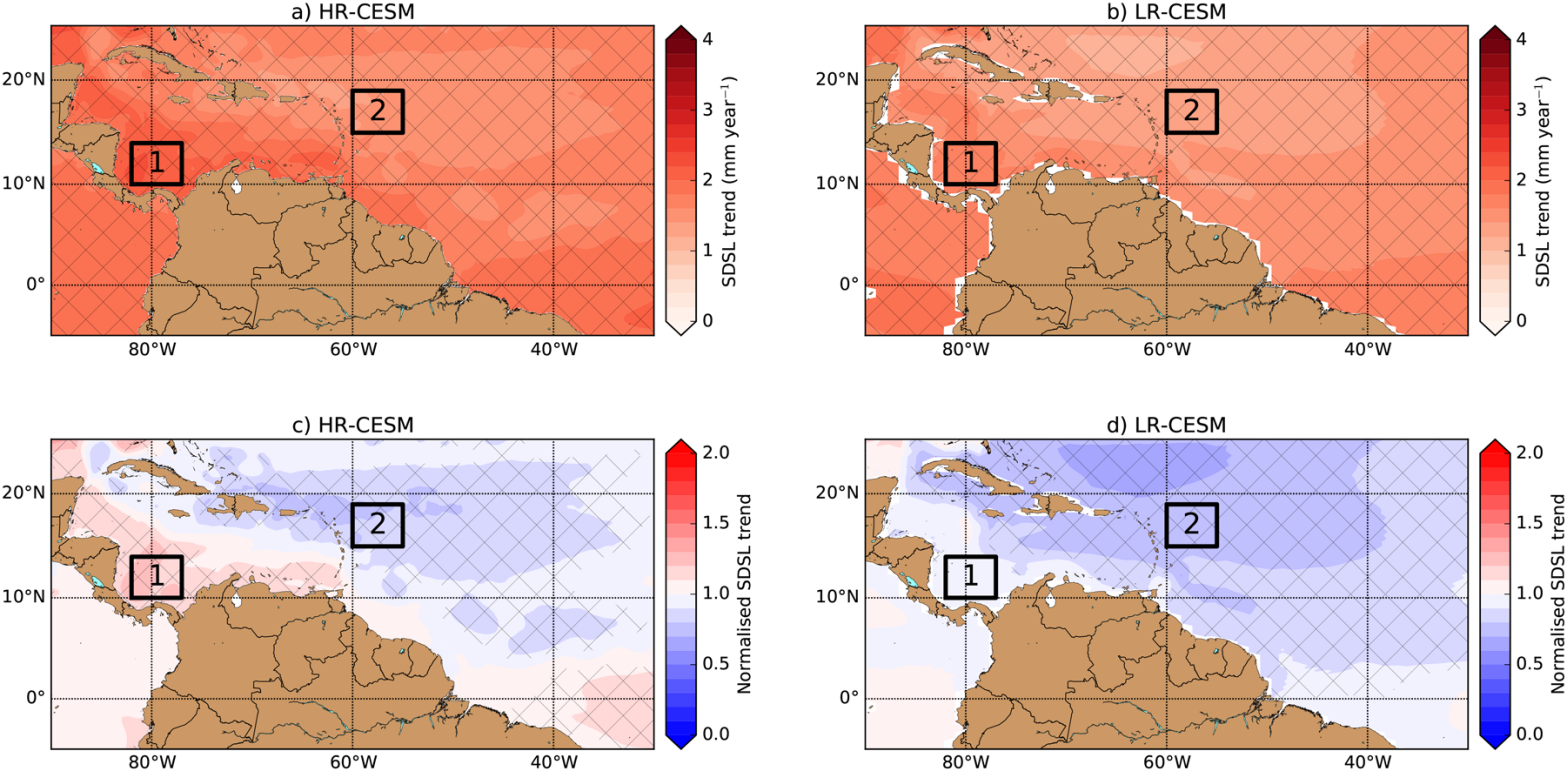
(**a**,**b**) Local SDSL ($$\eta$$) trend over the 101-year period for the HR- and LR-CESM. The hatched regions indicate significant ($$95\%$$-confidence level) trends. (**c**,**d**) The normalised SDSL trends (with respect to the $$\eta ^g_S$$ trend, Table 1) for the HR-CESM and LR-CESM. The hatched regions indicate a significantly ($$95\%$$-confidence level) different trend with respect to the $$\eta ^g_S$$ trend. In the panels two regions are defined, region 1 (south-west Caribbean Sea) is the domain $$10^\circ \,\hbox {N}$$–$$14^\circ \,\hbox {N}$$$$\times$$$$77^\circ \,\hbox {W}$$–$$82^\circ \,\hbox {W}$$ and region 2 (north-east of Caribbean Sea) is the domain $$15^\circ \,\hbox {N}$$–$$19^\circ \,\hbox {N}$$$$\times$$$$65^\circ \,\hbox {W}$$–$$70^\circ \,\hbox {W}$$, which will be used in subsequent analysis.

We first determined the trends in the yearly-mean SDSL field (indicated by $\eta$, see “Methods”) over the 101-year period (2000–2100) for the HR-CESM and LR-CESM simulations. The local SDSL change consists of two components: ocean dynamic sea-level change (indicated by $\eta _M$, DSL change) and global-mean thermosteric sea-level rise (indicated by $\eta _S^g$)^22^. The $\eta ^g_S$ of the HR-CESM and LR-CESM are corrected for any drift in their control simulations and are fairly similar when comparing the HR-CESM and LR-CESM (see Table 1; Supplementary Figure S7). The largest contribution ($>80\%$) to the total $\eta ^g_S$ originates from the upper 1,000 m of the ocean. Both simulations show a positive (and significant) SDSL trend over the displayed Caribbean region (Fig. 1a,b) and differences become more pronounced when these trends are normalised by the trend in $\eta ^g_S$ (Fig. 1c,d). The fastest SDSL rise occurs near the southern and western boundary of the Caribbean Sea in both simulations. However, the normalised SDSL trends in these parts of the Caribbean Sea are above average and below average with respect to the $\eta ^g_S$ trend for the HR-CESM and LR-CESM, respectively.

The differences in the (normalised) SDSL trends between the HR-CESM and LR-CESM are related to DSL changes. The local DSL ($\eta _M$) trend patterns are shown in Supplementary Figure S3a,b for the HR-CESM and LR-CESM, respectively, and there are large differences in both amplitude and sign (see also Supplementary Figure S4a). We also determined the local steric sea-level (indicated by $\eta _S$) change and the trends are shown in Supplementary Figure S3c,d. The local $\eta _S$ is corrected for any drift using the control simulations. The local $\eta _S$ trend is positive in the displayed region and the slowest $\eta _S$ trends are found on continental shelf for both CESM simulations. The $\eta _S$ trends in the Caribbean Sea and surroundings are faster in the HR-CESM compared to the LR-CESM (Supplementary Figure S4b).

The differences in the sign (above or below average) of the normalised SDSL trends across the Caribbean Sea (north–south or east–west) is also present in altimetry observations (Supplementary Figure S5). This dipole pattern in the normalised SDSL trends is related to the NBC and Caribbean Current, which typically determine the DSL distribution. The oceanic fronts and currents are much better represented in HR-CESM than LR-CESM^23^, leading to a different response in DSL in both simulations, of which the HR-CESM is more in agreement with observations.

Note that the observation record is much shorter (25 years) compared to the CESM simulations (101 years). Therefore we determined the normalised SDSL trend over a shorter period for the CESM simulations (Supplementary Figure S6). Here we present results of the normalised SDSL trends over both regions 1 and 2 (Fig. 1), which are situated west (region 1) and east (region 2) of the western boundary current. After model year 2050, the normalised SDSL trends have similar magnitudes and signs as the normalised SDSL trends over the entire period (2000–2100) and this results is robust for varying model initial year and the period over which the trends are determined. Before model year 2050, the magnitude and sign of the normalised SDSL trends are sensitive to the chosen period, which is likely related to natural variability as the simulations are initiated from a control simulation. The normalised SDSL centennial trends are representative for the shorter 25-year period (same length as observations) normalised SDSL trends in the second half of the CESM simulations, when the simulations are not in equilibrium. Both the present-day climate and the global-mean sea level are not in equilibrium either^6,8^. It is possible that observations shows persistent above-average and below-average sea-level trends for region 1 and region 2, respectively, due to natural variability, but the observational record is too short to falsify this hypothesis.

The time evolution of the DSL ($\eta _M$) and SDSL ($\eta$) averaged over both regions 1 and 2 are shown, together with the global-mean thermosteric sea-level rise ($\eta _S^g$), in Supplementary Figure S7. The DSL trends substantially differ for both HR-CESM and LR-CESM simulations. For example, the $\eta _M$ trends over region 1 are $0.37 \pm 0.05\,\hbox {mm}\,\hbox {year}^{-1}$ (HR-CESM, $99\%$-confidence Level (= CL)) and $-0.12 \pm 0.04\,\hbox {mm}\,\hbox {year}^{-1}$ (LR-CESM, $89\%$-CL). North east of the Caribbean Sea, in region 2, the $\eta _M$ trends are more similar with values $-0.32 \pm 0.05\,\hbox {mm}\,\hbox {year}^{-1}$ (HR-CESM, $99\%$-CL) and $-0.44 \pm 0.07\,\hbox {mm}\,\hbox {year}^{-1}$ (LR-CESM, $99\%$-CL). In the following section, we address what drives the long-term variability in the $\eta _M$ fields.

## Changes in the large-scale ocean circulation

Long-term variations in the $\eta _M$ fields are related to variability in the large-scale ocean circulation. For example, a weaker western boundary current results in a decrease in the zonal pressure gradient according to the geostrophic balance. Locally, the decreased pressure gradient leads to an increase (decrease) of $\eta _M$ west (east) of the western boundary current.

**Figure 2:**
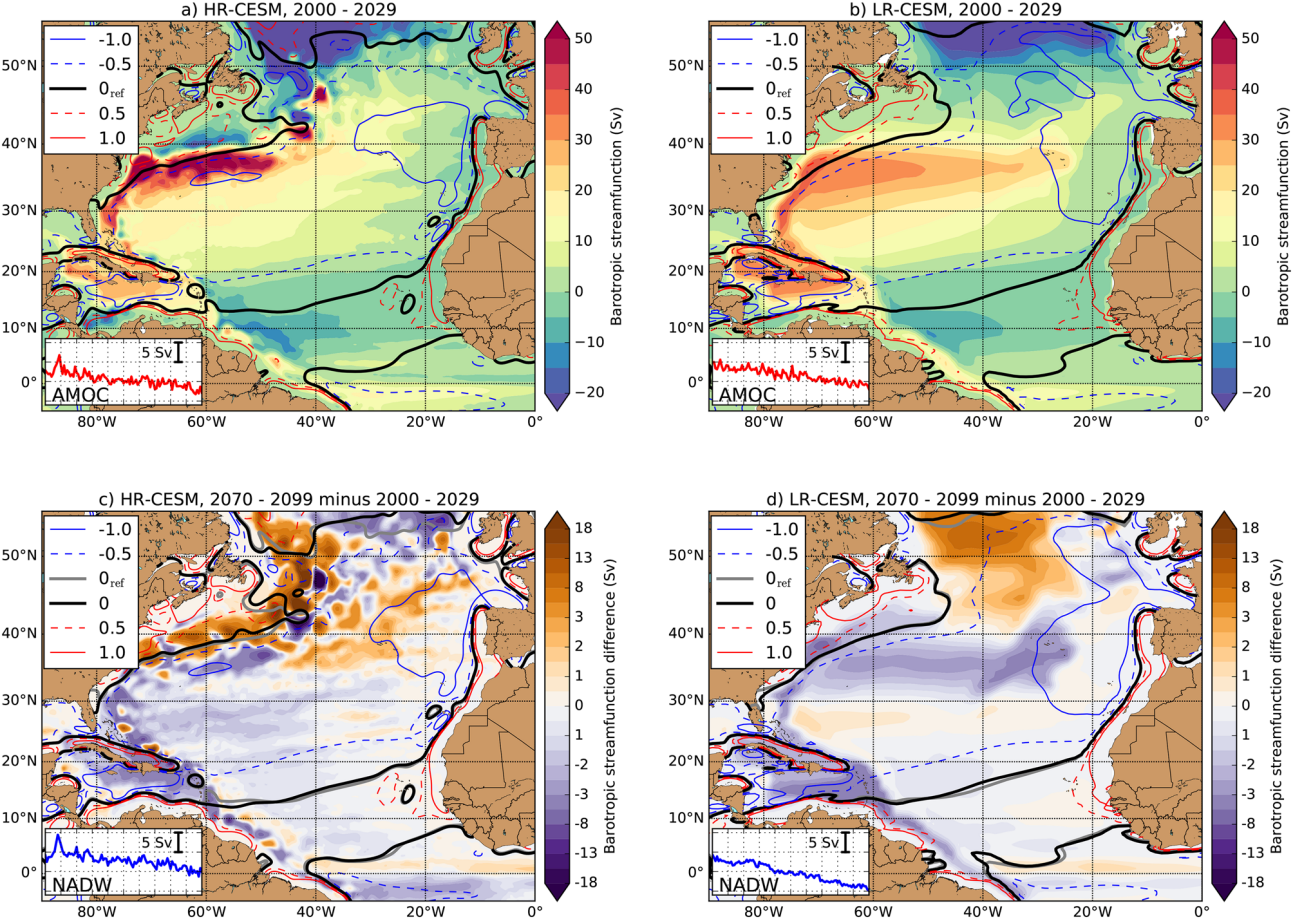
(**a**,**b**) Time mean over years 2000 – 2029 of the barotropic streamfunction (BSF) for the HR-CESM and LR-CESM. (**c**,**d**) Difference in BSF for the HR-CESM and LR-CESM between the time mean over years 2070–2099 and time mean over years 2000–2029. The contours show equal values of wind stress curl over (**a**,**b**): years 2000–2029 and (**c**,**d**): years 2070–2099, so not the difference over both periods, each spaced by 0.5 Pa per $10^4$ km, where the red (blue) contours indicate positive (negative) wind stress curl. The black contour indicates the 0 wind stress curl, where $0_{\mathrm {ref}}$ indicates the time mean over years 2000–2029. The insets show the AMOC- and NADW strength over the 101-year period, vertical range is between 9 and 26 Sv (1 Sv $\equiv 10^6\,\hbox {m}^3\,\hbox {s}^{-1}$, see “Methods” and the information in Supplementary Figure S10).

One way to determine changes in the large-scale circulation (which affects the $\eta _M$ fields) is by analysing the vertically integrated flow, represented by the barotropic streamfunction (BSF). The BSF is computed by meridionally integrating the zonal component of the vertically integrated flow, see “Methods”. The time-mean BSF fields at the beginning (model years 2000–2029) of both simulations are shown in Fig. 2a,b. The large-scale patterns of the subtropical gyre and subpolar gyre in the North Atlantic are represented in both CESM simulations. However, there are some notable differences in the BSF such as the Gulf Stream, which is better represented in the HR-CESM^24^ as reflected by the sharper BSF gradient (around $40^{\circ }\,\hbox {N}$, see Fig. 2a) compared to the LR-CESM (Fig. 2b).

The difference between the BSF over the years 2070–2099 and the years 2000–2029 is shown in Fig. 2c,d. Both the subtropical gyre and the subpolar gyre become weaker over time, with the largest negative BSF anomalies and meridional velocity anomalies occurring near western boundary currents (see also Supplementary Figure S8). Note the relatively large BSF anomalies in HR-CESM near $40^{\circ }\,\hbox {N}$. Small changes in the path of the Gulf Stream^25^ result in relatively large changes in BSF due to the sharp BSF gradients in that region. In LR-CESM, the BSF decreases almost over the entire Caribbean Sea and the BSF anomaly has a similar pattern as the normalised SDSL trends (Fig. 1d). We find significant correlations between the yearly-averaged $\eta _M$ and BSF fields in the Caribbean Sea over the 101-year period in the HR-CESM Control and LR-CESM Control (Fig. S9).

Long-term changes in BSF affect the $\eta _M$ fields according to geostrophic balance. The large-scale reduction in the BSF over time could in principle be related to changes in the vorticity input by the wind (i.e., the wind stress curl), but we find no substantial differences in the wind stress curl between the early and later period of the simulations (compare contours in Fig. 2a,c and Fig. 2b,d). We also did not find any reduction of the wind stress curl at the north coast of South America which potentially could explain the positive $\eta _M$ trends in this part of the Caribbean Sea^26^.

A change in the Atlantic Meridional Overturning Circulation (AMOC)^20,21,27^ can also affect the North Brazil Current and Caribbean Current strength^28,29^, since both currents are part of the northward branch of the AMOC. We find a significant decrease in the AMOC strength (see “Methods”), as seen through the insets in Fig. 2a,b and Supplementary Figure  S10. The southward branch of the AMOC [(i.e. the North Atlantic Deep Water (NADW), see “Methods”] also decreases over time (insets in Fig. 2c,d, Supplementary Fig. S10). Hence changes in Atlantic Ocean circulation captured by the BSF, in particular those in the NBC, are mainly related to a reduction of the AMOC strength and consequently affect the $\eta _M$ fields in the Caribbean Sea.

## Future sea-level extremes

**Figure 3:**
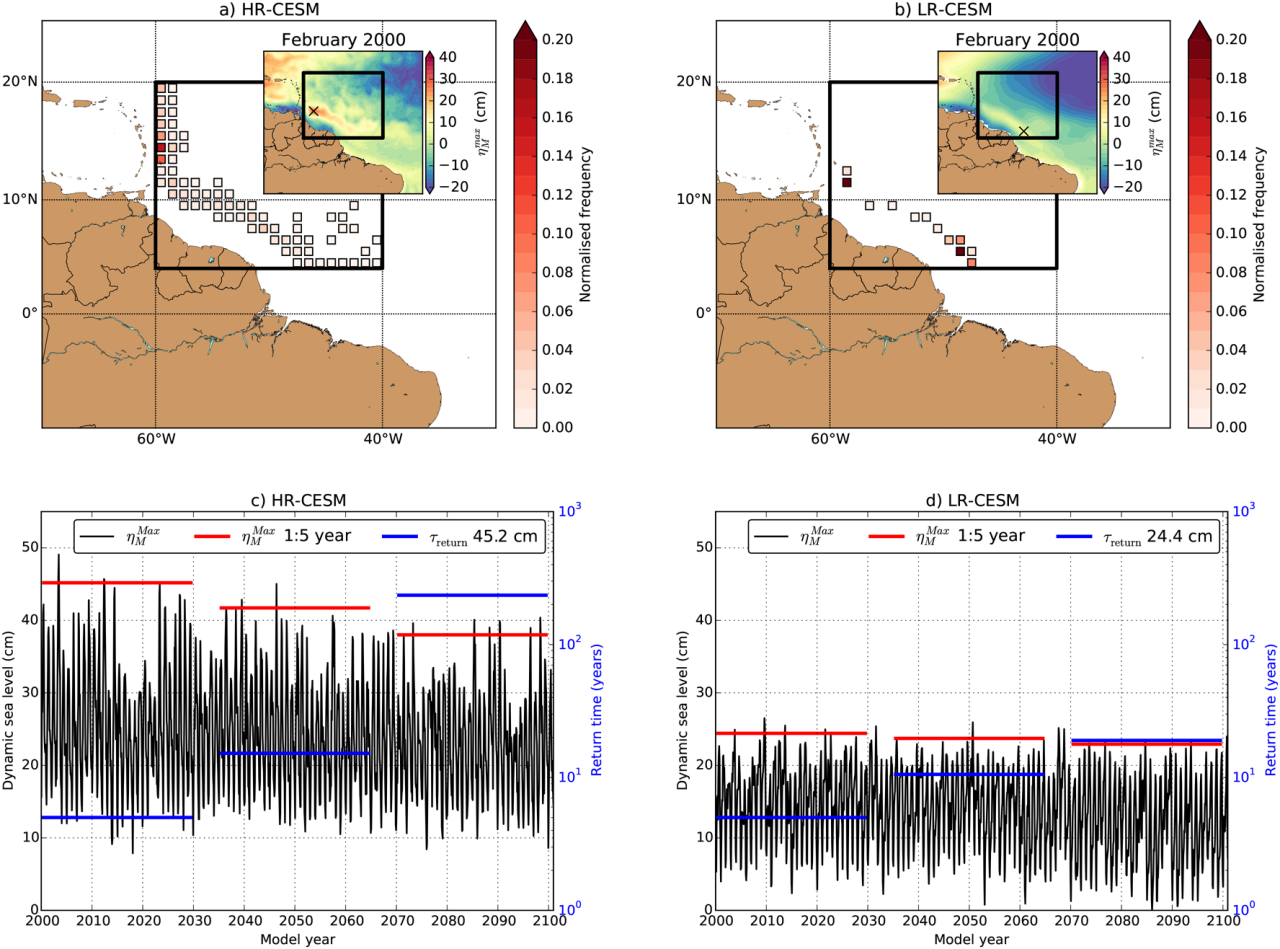
(**a**,**b**) Histogram of the location of $$\eta _M^{Max}$$ over the NBC outflow region (black outlined region, $$4.5^\circ \,\hbox {N}$$–$$20^\circ \,\hbox {N}$$$$\times$$$$40^\circ \,\hbox {W}$$–$$60^\circ \,\hbox {W}$$) for the HR-CESM and LR-CESM simulations. The inset shows the $$\eta _M^{max}$$ field for February, model year 2000, including the location (cross) of the maximum $$\eta _M^{Max}$$ in the NBC outflow region. (**c**,**d**) Monthly time series of $$\eta _M^{Max}$$ (black curve). The red lines are the expected $$\eta _M^{Max}$$ which occur once every 60 months (1:5 year event) for three periods (model years 2000–2029, 2035–2064 and 2070–2099). The blue lines are the return time for a fixed $$\eta _M^{Max}$$ of 45.2 cm (HR-CESM) and 24.4 cm (LR-CESM), which is based on the 1:5 year event of the first 30 years (model years 2000–2029).

A prominent feature of the NBC is its retroflection which sheds off multiple anti-cyclonic eddies per year. These NBC eddies propagate along the background flow towards the Lesser Antilles and sometimes (partially) enter the Caribbean Sea^30^. To determine the effect of the NBC eddies (which are shed by the NBC retroflection) on the DSL, we analyse the local maxima of $\eta _M$. The $\eta _M$ signature of the NBC eddies is partly filtered out in the monthly-averaged (or yearly-averaged) $\eta _M$ fields due to the time averaging. Therefore, we analyse daily-averaged $\eta _M$ fields over the 101-year period for both HR-CESM and LR-CESM simulations. Using the daily-averaged $\eta _M$ fields, we determine the local monthly maximum of $\eta _M$ (indicated by $\eta _M^{max}$) for both simulations (insets in Fig. 3a,b). The cross here indicates the location of the maximum value, $\eta _M^{Max}$, over the NBC outflow region ($4.5^\circ \,\hbox {N}$ – $20^\circ \,\hbox {N} \times 40^\circ \,\hbox {W}$ – $60^\circ \,\hbox {W}$), indicated as the black outlined region in Fig. 3a,b. The distribution of $\eta _M^{Max}$ over the 101-year period is also shown in Fig. 3a,b for both simulations.

In the HR-CESM, the path along which the NBC eddies propagate is clearly represented in the $\eta _M^{Max}$ distribution; this path is obviously absent in the LR-CESM. The time series of $\eta _M^{Max}$ retained from the $\eta _M^{Max}$ distribution, and hence each maximum can occur at different locations, has an overall higher time mean for the HR-CESM (Fig. 3c) compared to the LR-CESM (Fig. 3d). The extreme statistics of the $\eta _M^{Max}$ time series is analysed with Generalised Extreme Value (GEV) techniques^31^, see Methods, and the GEV fits are shown in Supplementary Figure S11. The level of $\eta _M^{Max}$ which returns once every 5 years (a 1:5 year moderately extreme event) decreases by 7.2 cm ($-16\%$) at the end of the simulation for the HR-CESM. For the LR-CESM, this decrease in the 1:5 year event is only 1.5 cm ($-6\%$). We found a similar decrease in $\eta _M^{Max}$ when taking the yearly maxima (removing the seasonal cycle) but the yearly maxima GEV fits are less robust due to fewer data points (30 instead of 360).

Similar to the monthly $\eta _M^{max}$ fields, we also determined the yearly $\eta _M$ maximum fields (retained from daily averages) and trends over the 101-year period for the simulations (see Supplementary Figure S12a,b). In the NBC outflow region, this trend in the yearly $\eta _M$ maximum is three to four times larger than the trend in the yearly-averaged $\eta _M$ (cf. Supplementary Figure S3c,d) for the HR-CESM (Supplementary Figure S12c). For the LR-CESM, the trend in the yearly $\eta _M$ maximum is only twice the magnitude of the yearly-averaged $\eta _M$ trend in the NBC outflow region (Supplementary Figure S12d).

Apart from the $\eta _M$ changes, we also find a weakening of the horizontal surface velocities in the NBC transport region (Supplementary Figure S13). Both HR-CESM and LR-CESM simulations show a path of significant ($95\%$-confidence level) negative velocity trends from the NBC retroflection to the Lesser Antilles. This path of decreasing velocities in the LR-CESM is much broader compared to that in the HR-CESM.

To summarise, the differences in $\eta _M$ extremes and horizontal velocities in the NBC outflow region between the HR-CESM and LR-CESM simulations are related to the horizontal resolution of the models. In the LR-CESM simulation, the NBC retroflection and shedding of NBC eddies are parameterised and the relevant mesoscale dynamics in this region is not captured^11^. At least in the NBC outflow region, DSL projections based on the LR-CESM can result in overestimations in the 1:5 year moderately extreme event (over a period of 100 years) compared to the HR-CESM, with about a 6 cm (factor five) difference.

## Sterodynamic sea-level trends in CMIP6 models

Most models participating in the Coupled Model Intercomparison Project phase six (CMIP6, see “Methods”) do not explicitly capture mesoscale processes as they have a similar horizontal resolution as in the LR-CESM simulation (i.e., $1^{\circ }$). An analysis of the CMIP6 output (under the 1% $\hbox {pCO}_2$ increase scenario) shows an increase in $\eta _S^g$ and a decrease in the AMOC strength over the simulated period, which is a similar response as in our CESM simulations (Supplementary Figure S14). In the CMIP6 models, there is a wide range in SDSL trends in the Caribbean Sea over a 101-year period (Supplementary Figure S15). The horizontal velocity trends (Supplementary Figure S16) are similar to our LR-CESM simulation, even for the eddy-permitting models (i.e. $0.25^{\circ }$).

**Figure 4:**
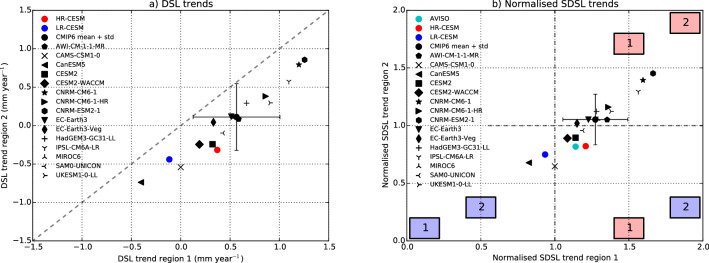
Linear trends over the 101-year period of (**a**): $\eta _M$ and (**b**): normalised $\eta$ [with the $\eta ^g_S$ trend, see Table 1) for the HR-CESM, LR-CESM, the CMIP6 output and (in **b**)] AVISO for the two different regions 1 and 2 (cf. Fig. 1). In (**b**), the AVISO determined observations (the total sea level, $\eta$) are normalised with a value of 3 mm year$^{-1}$. The colour-coded boxes in the quadrants indicate above-average (red) and below-average (blue) SDSL trends with respect to the $\eta ^g_S$ trend for the two regions.

Figure 4b indicates that, from comparing the normalised SDSL trends (with respect to the $\eta ^g_S$ trend) over regions 1 (south-west Caribbean Sea) and 2 (north-east of Caribbean Sea), the HR-CESM has the same normalised SDSL signs as observations for both regions (i.e. in the same quadrant). Note that the observed normalised sea-level trends (by $3\,\hbox {mm}\,\hbox {year}^{-1}$^8^) include all contributions of sea-level rise, so it is not the ‘pure’ SDSL trend. The contribution of melt by the Greenland- and the Antarctic ice sheets is distributed homogeneously over the Caribbean region^9^ and will not affect the sign of the observed normalised sea-level trends (it will change the numerical values). Ten (Two) out of the fifteen CMIP6 models show positive (negative) DSL trends for both regions (Fig. 4a). Only three CMIP6 models have the same normalised SDSL signs as observations for both regions (Fig. 4b). The normalised SDSL CMIP6 mean and standard deviation is $1.27\,\pm \,0.22$ and $1.05\,\pm \,0.22$ for region 1 and region 2, respectively. The normalised sea-level trend in observations is 1.14 and 0.82 for region 1 and region 2, respectively. Note that the observed trends are determined over a shorter period compared to the centennial CESM and CMIP6 trends, as already discussed in Supplementary Figure S6. The CMIP6 mean does not have the same SDSL sign as observations for region 2. The normalised SDSL of the HR-CESM (as well as the LR-CESM) lies outside the CMIP6 standard deviation for region 2. This mismatch for region 2 is related to the misrepresentation of the effects of NBC eddies, which are not captured by the CMIP6 models. From these preliminary CMIP6 model results, the largest biases in SDSL are found in region 2.

## Conclusions and implications

By analysing model simulations from high- (HR-CESM) and low-resolution (LR-CESM) versions of the Community Earth System Model (CESM) over a 101-year period under a greenhouse gas forcing scenario, we have shown substantially different responses in the dynamic sea level (DSL, $\eta _M$) in the Caribbean Sea and its surroundings.

Heat uptake by the (upper) ocean causes global-mean thermosteric sea-level rise ($\eta _S^g$), which is the main contribution of local sterodynamic sea-level rise (at least $75\%$). Although the effects of ice-sheet mass loss and isostatic adjustment are not taken into account in any of the presented model simulations, these are expected to be fairly homogeneous on the regional scale^9,32^ and can be incorporated in the global-mean thermosteric sea-level rise (e.g. in $\eta ^g_S$ in this study).

The changes in $\eta _M$ are mainly related to a reduced Atlantic Meridional Overturning Circulation (AMOC) and the associated changes in the North Brazil Current (NBC). In the HR-CESM simulation, the reduced strength of the NBC results in a decreased eddy activity and strength, resulting in weaker $\eta _M$ extremes. For the 1:5 year moderately extreme event in the maximum of $\eta _M$ over the NBC region, there can be a factor 5 difference in DSL values between HR-CESM and LR-CESM.

In the Caribbean Sea and surroundings, mesoscale processes (such as the NBC retroflection and eddies) are important for regional sea-level projections. Adequate Caribbean sea-level projections can only be obtained in ocean models which capture mesoscale oceanic processes. Consequently, most CMIP6 models as well as the LR-CESM are not fit for purpose in making such projections. The higher resolution ocean models (AWI-CM-1-1-MR and CNRM-CM6-1-HR) of the CMIP6 protocol with a $0.25^\circ$ ocean model resolution^33^, are not noticeably better compared to the coarse CMIP6 models in the Caribbean region.

We have shown that the effect of DSL can locally have a large contribution to the sterodynamic sea-level change. The DSL is affected by mesoscale processes everywhere, but particularly in eddy-active regions such as near western boundary currents. Hence, although we only showed results for the Caribbean, the effects of ocean model horizontal resolution will be important for sea-level projections in other regions and needs to be further investigated. Due to the high-computational costs, only one realisation of the high-resolution model was available, yet the results here indicate that an ocean model with a $0.1^\circ$ horizontal resolution agrees much better to observations and this appears to be the minimum resolution required to capture an adequate dynamic sea-level field.

For the Caribbean region, the good news is that changes in dynamic sea-level extremes in the high-resolution model projects a much lower sea-level rise than the low-resolution model, in particular for the many island areas in the eastern part of the region. High-resolution climate models are crucial for a better understanding of future sea-level rise and sea-level extremes, and for planning future investments to adapt to effects of climate change in the Caribbean region and elsewhere over the globe.

## Methods

### Climate model simulations with CESM

In this study, we use model output of the Community Earth System Model (CESM) version 1.04. The high-resolution coupled climate version^34^ has an ocean component and sea-ice component with a $0.1^{\circ }$ horizontal resolution on a curvilinear, tri-polar grid which captures the development and interaction of mesoscale eddies^11^. The ocean model (the Parallel Ocean Program) has 42 non-equidistant depth levels, with the highest vertical resolution near the surface. The atmosphere- and land surface components have a horizontal resolution of $0.5^{\circ }$, and the atmosphere component has 30 non-equidistant pressure levels.

We analyse two simulations of the high-resolution CESM which are initiated after a spin-up period of 200 years. The spin-up of the CESM has fixed forcing conditions ($\hbox {CO}_2$, methane, solar insolation, aerosols) of the year 2000 which are repeated every year. The two simulations consists of a control simulation (extension of the spin-up) and a forced simulation with a prescribed increase of atmospheric $\hbox {CO}_2$ levels (2000–2100) which is retained from the Representative Concentration Pathways (RCP) 8.5 scenario^35^. The forced simulation is similar to a $1\%$ increase of $\hbox {pCO}_2$ each year. Both simulations have a 101 years’ duration (model years 200–300).

Supplementary Figure S1 shows some key variables for the spin-up period (similar results as in^36^), the control simulation and the forced simulation for the high-resolution model. For the control simulation, the global mean (2 m) surface temperature is almost constant and the radiative imbalance is slightly positive. The Gregory plot (Supplementary Figure S1c) also shows the equilibration of the control simulation and the deviation from the equilibrium for the forced simulation. The upper 700 m global ocean heat content (Supplementary Figure S1d) is still adjusting, but relatively small trends (compared to the spin-up) occur over the last 100 years. The deep ocean fields take a much longer time to equilibrate. In the forced simulation, the surface temperature, radiative imbalance and ocean heat content start to deviate from the control simulation due to the increase of atmospheric $\hbox {CO}_2$ concentration.

In addition to the high-resolution CESM simulations, a companion simulation was conducted at a lower resolution (using CESM version 1.1.2). This model’s ocean component has a horizontal resolution of $1^{\circ }$ and 60 non-equidistant depth levels. The atmospheric component has a horizontal resolution of $1^{\circ }$ and 30 non-equidistant pressure levels. The low-resolution simulation of the CESM had a spin-up period of 1,200 years with the same fixed forcing conditions as the high-resolution version. After the spin-up, we conduct the two simulations: one control simulation and one forced simulation, similar to the set-up of the high-resolution version. Both simulations have a 101 years’ duration (model years 1,200–1,300).

### Model output analysed

The standard model output of the CESM simulations consists of monthly averaged fields of sea surface height above geoid ($\eta _M$), horizontal velocity, temperature and salinity. All of the monthly averaged quantities are converted to yearly averages. The globally averaged $\eta _M$ fields are about zero since the ocean is volume conserving. A limited number of quantities, for example $\eta _M$, are available as daily averages for the CESM simulations and are used in generalised extreme value analysis.

### CMIP6 data

We use results from the latest release of the Coupled Model Intercomparison Project phase six (CMIP6) and compare these to the output of our CESM simulations (Table 1). We analysed the preliminary model output of the CMIP6 in which atmospheric $\hbox {CO}_2$ levels increase each year by $1\%$. Note that each model of the CMIP6 is initiated from the pre-industrial (year 1,850) control simulation. Only models which conserve ocean volume (as the CESM) are considered here (variable name ‘zos’). We analyse the monthly averaged $\eta _M$, horizontal velocity, temperature and salinity fields of the first 101 model years, as is done for the CESM output. The model output of the AWI-CM-1-1-MR is provided on an unstructured grid. Before analysing the output of this CMIP6 model, the data is interpolated onto the HR-CESM grid.

**Table 1:** Overview of the CMIP6 and CESM models with the resolution of the ocean grid.

Model	lon $\times$ lat (number of grid cells)	Nominal resolution (km)	Vertical layers	$\eta ^g_S$ trend (mm year$^{-1}$)	AMOC (Sv)
HR-CESM	$3600 \times 2400$	10	42	1.8	18.2
LR-CESM	$320 \times 384$	100	60	1.8	19.0
AWI-CM-1-1-MR	Unstructured	25	46	1.7	18.5
CAMS-CSM1-0	$360 \times 200$	100	50	1.5	12.7
CanESM5	$360 \times 291$	100	45	2.3	9.6
CESM2	$320 \times 384$	100	60	2.3	16.7
CESM2-WACCM	$320 \times 384$	100	60	2.2	16.9
CNRM-CM6-1	$362 \times 294$	100	75	2.0	14.4
CNRM-CM6-1-HR	$1442 \times 1050$	25	75	2.4	11.4
CNRM-ESM2-1	$362 \times 294$	100	75	1.9	16.3
EC-Earth3	$362 \times 294$	100	75	2.3	13.8
EC-Earth3-Veg	$362 \times 294$	100	75	2.2	13.0
HadGEM3-GC31-LL	$360 \times 330$	100	75	2.4	13.3
IPSL-CM6A-LR	$362 \times 332$	100	75	1.9	9.2
MIROC6	$360 \times 256$	100	63	1.8	14.9
SAM0-UNICON	$320 \times 384$	100	60	2.3	19.4
UKESM1-0-LL	$360 \times 330$	100	75	2.4	13.0

The global-mean thermosteric sea-level ($\eta ^g_S$) trend (101 years) and the time mean of the AMOC strength (at $26^{\circ }\hbox {N}$ and 1,000 m, see below) over the first 10 years of each simulation.

### Significance of the linear trends

The trends are derived from a linear least-square fit to the yearly-averaged time series. The significance of each trend is determined following the procedure outlined in^37^, while taking into account the reduction of degrees of freedom for the time series which are not statistically independent. First, the variance $s_e$ of the residuals of the linear fit (*e*(*t*)) are determined. The degrees of freedom are reduced using the lag-1 autocorrelation ($r_1$) of the residuals,

1 $$\begin{aligned} s_e^2 = \frac{1}{N \frac{1 - r_1}{1 + r_1} - 2} \sum _{t = 1}^N e(t)^2 \end{aligned}$$

where *N* is the number of years (i.e. $N = 101$ years). The standard error of the residuals, $s_b$ is

2 $$\begin{aligned} s_b = \frac{s_e}{\sqrt{\sum _{t = 1}^{N} \left( t - \frac{N + 1}{2} \right) ^2 }} \end{aligned}$$

The Student-*t* value is the ratio between the linearly fitted trend and the standard error. Using the reduced degrees of freedom and the two-sided critical Student-*t* values, one can determine the significance of having a trend different from zero (the null hypothesis).

### Sterodynamic sea level

The local sterodynamic sea level (SDSL) consists of two components^22^. The first component is the dynamic sea level ($\eta _M$) and is the height of the ocean surface above the geoid. The $\eta _M$ fields are part of the standard output in the CESM simulations (variable ‘SSH’) and CMIP6 models (variable ‘zos’). The $\eta _M$ fields have a global mean of about 0, if the global mean was non zero we subtracted uniformly the global mean from the $\eta _M$ fields.

The next component is the global-mean thermosteric sea-level rise ($\eta _S^g$), which is determined from post-processing the model output^38^. First, we determined the local steric sea-level ($\eta _S$). The contribution of both thermal changes and haline changes is determined as the full-depth integral over the specific volume anomaly^39^,

3 $$\begin{aligned} \eta _S = \int _{-H}^{0} \frac{\rho _0 - \rho (T, S, P)}{\rho _0} \mathrm {d}z \end{aligned}$$

The temperature, salinity and pressure dependency are taken into account while determining the density, and $\rho _0 = 1,028$ kg m$^{-3}$. The steric sea-level change is expressed as an anomaly with respect to the initial value of the first model year. The global-mean thermosteric sea-level ($\eta _S^g$) rise is determined by globally averaging $\eta _S$. This procedure is done for both the CESM simulations as the CMIP6 models. One can use the variable ‘zostoga’ instead of $\eta _S^g$ for the CMIP6 models, but this is not used in this study for comparison with the CESM simulations.

Eventually, hence the local sterodynamic sea level ($\eta$) is determined from

4 $$\begin{aligned} \eta = \eta _M + \eta _S^g \end{aligned}$$

The trend over the 101-year period for $\eta _M$ and $\eta _S$ are shown in Supplementary Figure S3. We corrected for any drift in $\eta _S$ and $\eta _S^g$ using the control simulations.

### Barotropic streamfunction

The barotropic flow is defined as the full depth integral of the horizontal velocity:

5 $$\begin{aligned} {\overrightarrow{BF}} = \int _{-H}^{0} \vec {v}\,\mathrm {d}z \end{aligned}$$

Starting from Antarctica (with a value of 0 for the barotropic streamfunction), we integrate the zonal component of the barotropic flow (indicated by $BF_x$) meridionally to determine the barotropic streamfunction:

6 $$\begin{aligned} \mathrm {BSF} = \int _{90^{\circ }\mathrm {S}}^{90^{\circ }\mathrm {N}} BF_x\,\mathrm {d}y \end{aligned}$$

For convenience, the average value of the BSF along the African coast line is subtracted from the entire BSF field.

### AMOC strength (definition)

The AMOC strength is defined as the total meridional mass transport at $26^{\circ }\,\hbox {N}$ integrated between $80.5^{\circ }\,\hbox {W}$ and $12^{\circ }\,\hbox {W}$ (RAPID array) and integrated over the upper 1,000 m:

7 $$\begin{aligned} \mathrm {AMOC} = \int _{-1000}^{0} \int _{80.5^{\circ }\mathrm {W}}^{12^{\circ }\mathrm {W}} v\,\mathrm {d}x \mathrm {d}z \end{aligned}$$

Observations show that the AMOC strength (3-month average) at $$26^{\circ }\,\hbox {N}$$ varies between 10 and 24 Sv (1 Sv $$\equiv$$$$10^6\,\hbox {m}^3\,\hbox {s}^{-1}$$), with a mean transport of about 16–19 Sv^21,40^.

### NADW strength (definition)

The NADW strength is defined as the total meridional mass transport at $40^{\circ }\,\hbox {N}$ integrated between $77^{\circ }\,\hbox {W}$ and $8^{\circ }\,\hbox {W}$ and between 1500 and 4000 m.

8 $$\begin{aligned} \mathrm {NADW} = \int _{-4000}^{-1500} \int _{77^{\circ }\mathrm {W}}^{8^{\circ }\mathrm {W}} v\,\mathrm {d}x \mathrm {d}z \end{aligned}$$

### Generalised extreme value

The $\eta _M$ extremes in the NBC outflow region are analysed using GEV (Generalised Extreme Value) analysis. First, we retained the monthly $\eta _M$ maxima ($\eta _M^{Max}$) from daily averaged $\eta _M$. This is the block maxima GEV approach where each month contains the $\eta _M$ maximum. The distribution of $\eta _M^{Max}$ may vary in time. Therefore different periods of *n* months’ length are retained from the full time series, under the assumption that these periods in time are stationary and stationary GEV analysis can be conducted^41^. Using all the data points over a particular period, a GEV fit is made to the data points using the following expression as the cumulative distribution function:

9 $$\begin{aligned} G_n(z) = \exp \left( - \left( 1 + \xi \left( \frac{z - \mu }{\sigma } \right) \right) ^{-1 / \xi } \right) \end{aligned}$$

where $\mu$, $\sigma$, $\xi$ are the location, scale and shape parameter, respectively. A special case of the distribution is where $\xi \rightarrow 0$, the GEV distribution approaches a Gumbel distribution. The probability of occurrence (*p*) using the distribution of $G_n(z)$ is related to return time ($\tau$) of such an event:

10 $$\begin{aligned} \tau = \frac{1}{p} \end{aligned}$$

The value of $\eta _M^{Max}$ related to this probability ($z_p$) can be obtained by:

11 $$\begin{aligned} z_p = \mu - \frac{\sigma }{\xi } \left( 1 - \left( - \log \left( 1 - p \right) \right) ^{-\xi } \right) \end{aligned}$$

Note that detrending the periods of time (linear or quadratic) might result in biases. Therefore, the data on which GEV is applied is directly fitted to the $G_n(z)$ distribution. We have chosen to fit the $G_n(z)$ distribution using periods of 360 months’ length (30 years). Shorter sections (120 months) provided similar results, but might have insufficient data to make a reasonable fit.

## Supplementary information

Supplementary material 1

## Data Availability

All the model output is analysed with Python 2.7.9. The maps are generated using the Basemap package in Python. All figures are prepared with Python 2.7.9. Part of the Python code as well as processed CESM and CMIP6 model output can be accessed at https://github.com/RenevanWesten/SR-Caribbean.
