# Supplementary material for: Ocean model resolution dependence of Caribbean sea-level projections
Source: Sci Rep. 2020 Sep 3;10:14599. doi: 10.1038/s41598-020-71563-0 (PMC7471300; doi:10.1038/s41598-020-71563-0)
Supplement: Supplementary file 1 — Supplementary material 1 [file 41598_2020_71563_MOESM1_ESM.pdf]

Supplementary Figures

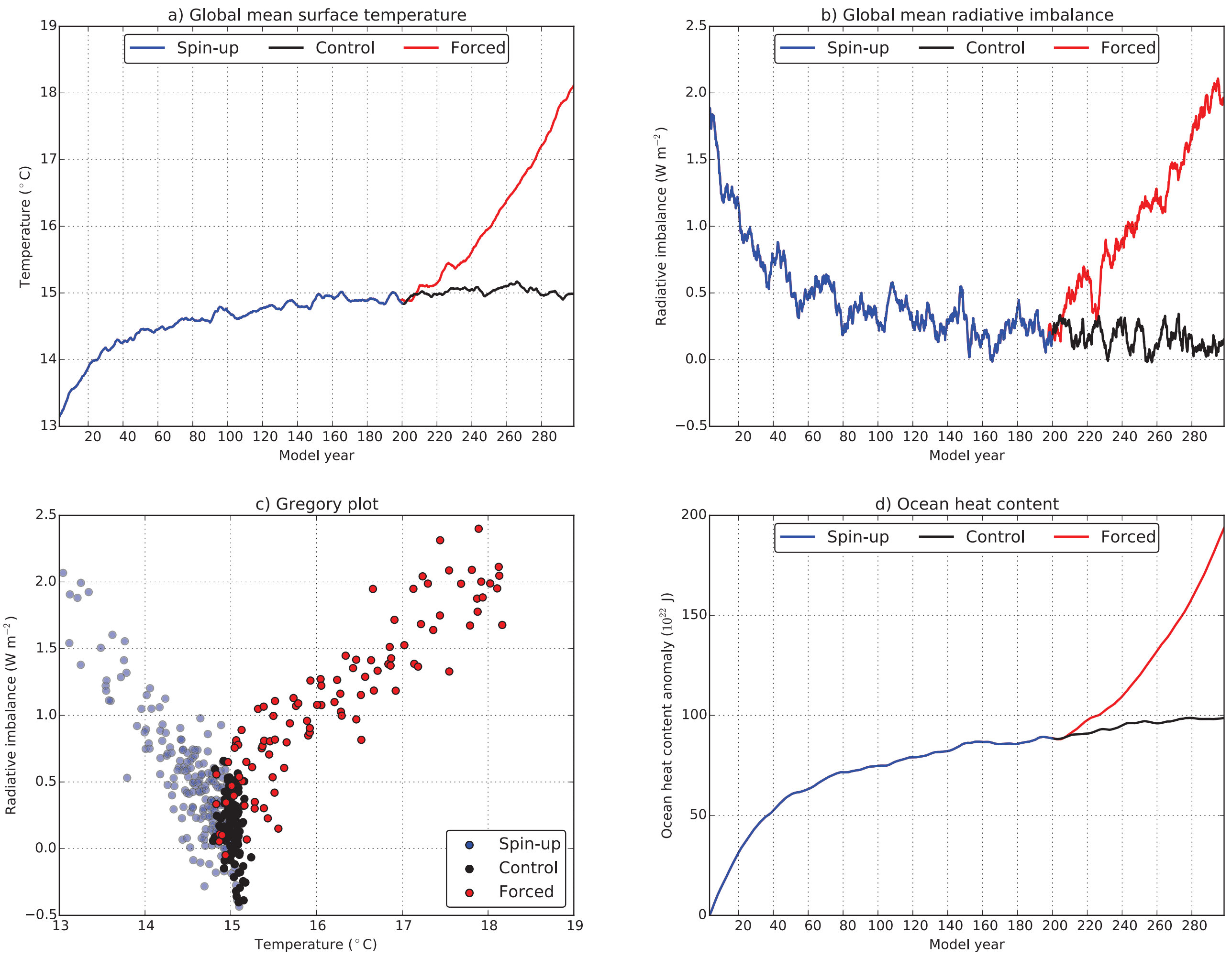

Figure S1. Equilibration and forcing of the HR-CESM simulations: (a): the global mean 2-meter surface temperature, (b): the global radiative imbalance at the top of the model, (c): Gregory plot of the yearly averaged surface temperature and radiative imbalance and (d): the global ocean heat content anomaly of the upper 700 m with respect to the initial value (2.5×10^{26} J at model year 1). The blue, black, and red curves (or points) represent the Spin-up, HR-CESM Control and HR-CESM simulations, respectively. All time series in a), b) and d) are smoothed by a 60-month running mean.

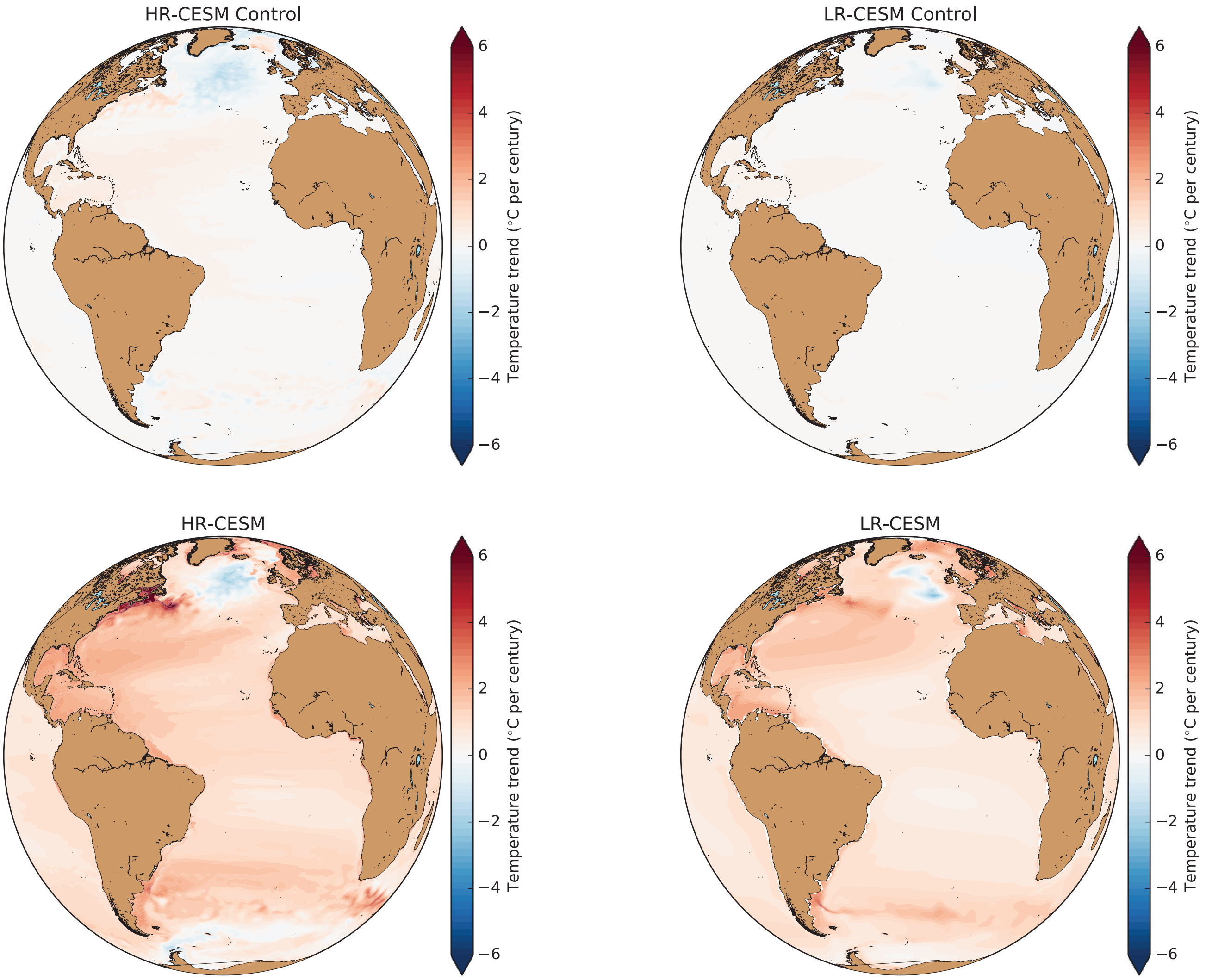

Figure S2. Upper 1000 m temperature trend over the 101-year period for the CESM simulations.

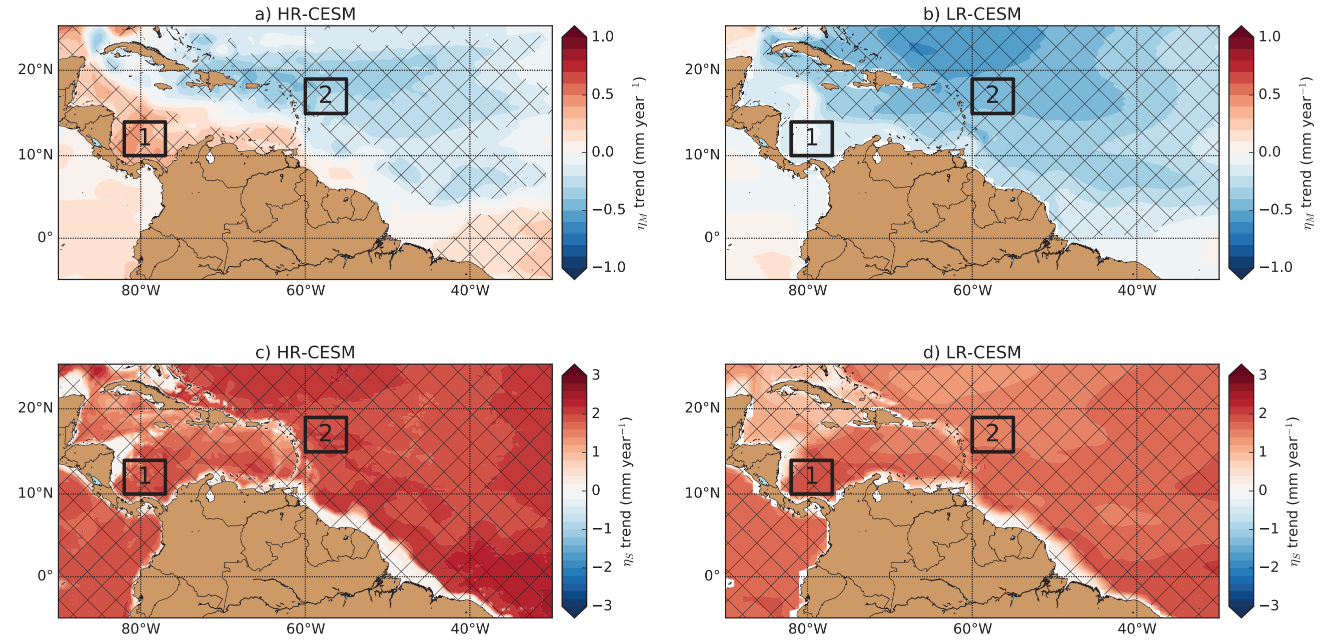

Figure S3. (a & b): Local dynamic sea-level (η_M) trend and the (c & d): local steric sea-level (η_S) trend over the 101-year period for the HR-CESM and LR-CESM. The hatched regions indicate significant (95%-confidence level) trends. The regions 1 and 2 are the same as in Figure 1. The local η_S are corrected for any drift in the HR-CESM Control and LR-CESM Control simulations.

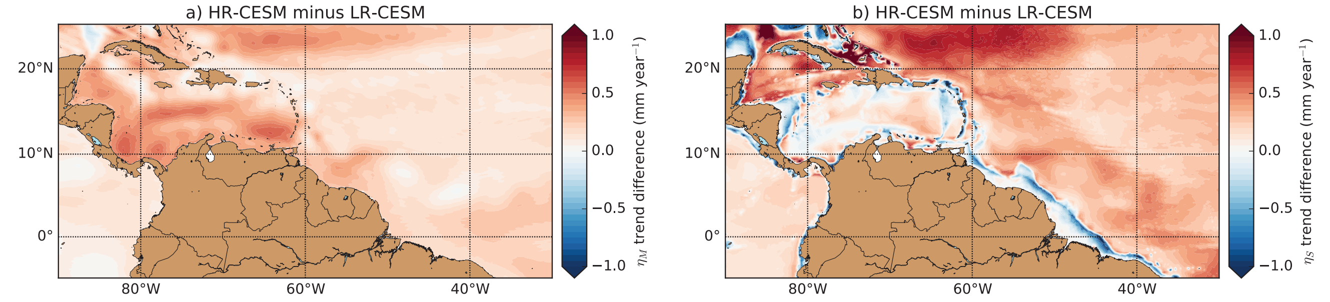

Figure S4. Pattern difference between the HR-CESM and LR-CESM for the (a): η_M trend and (b): η_S trend. Before determining the difference, the LR-CESM is interpolated onto the HR-CESM grid.

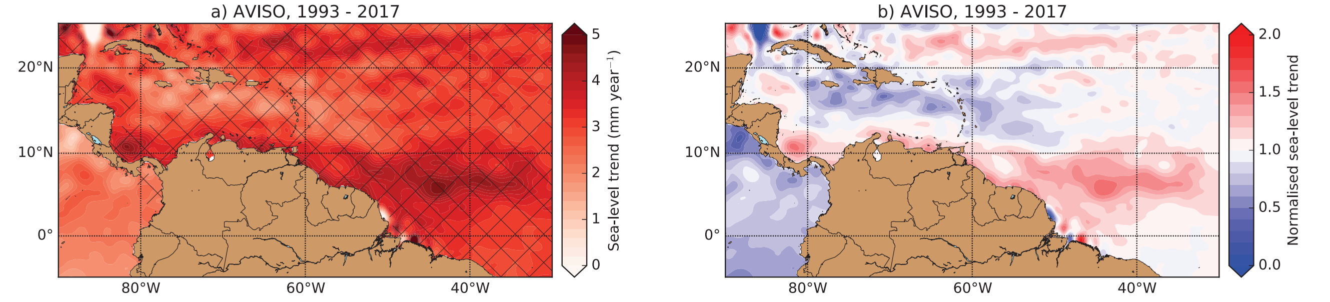

Figure S5. (a): Local sea-level trend from the AVISO (Archiving, Validation and Interpretation of Satellite Oceanographic data base observations) between 1993 – 2017. The hatched regions indicate significant (95%-confidence level) trends. (b): Local sea-level trend normalised to the global sea-level trend (= 3 mm year^{-1},[8]).

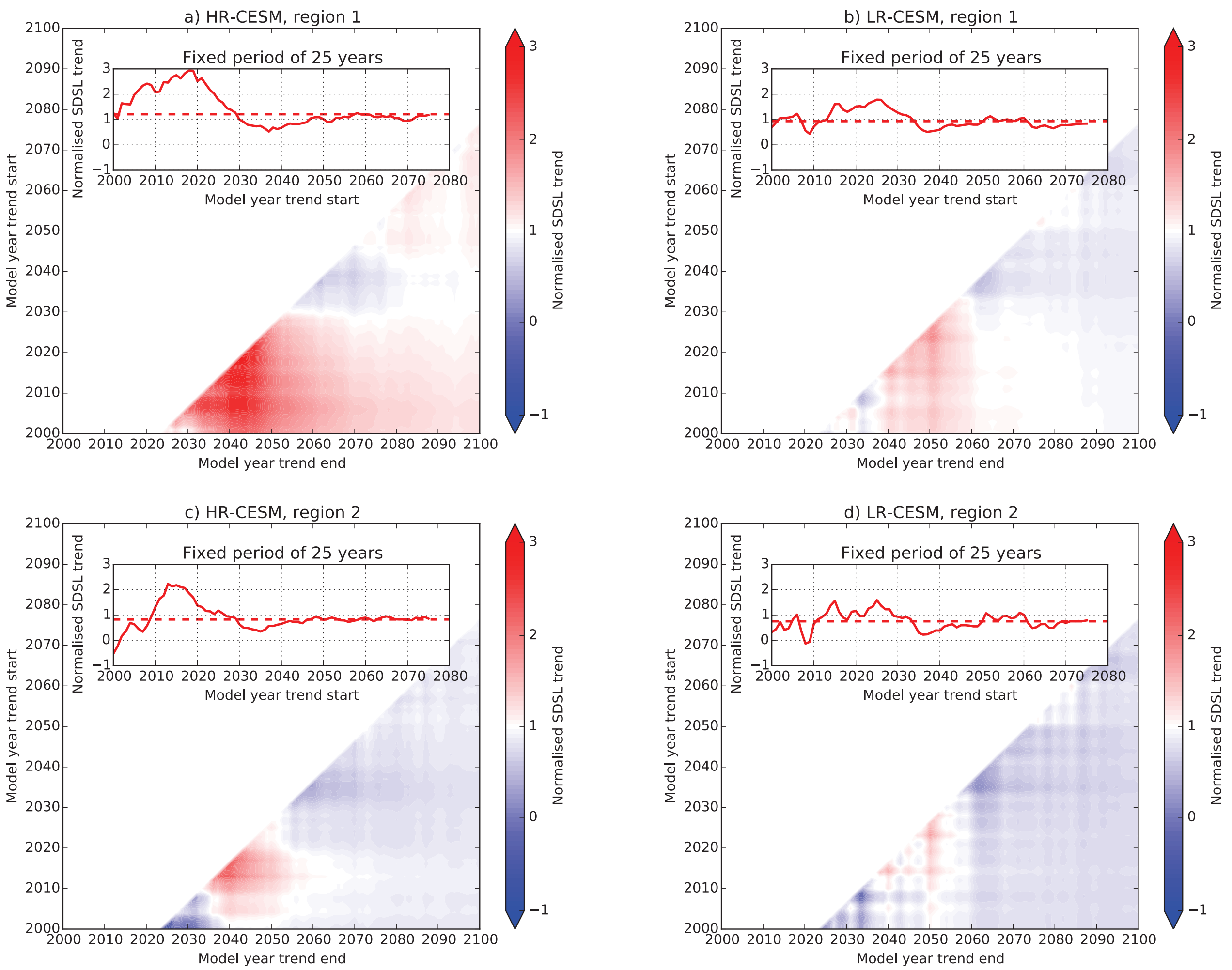

Figure S6. The normalised SDSL trend (w.r.t. η_S^g) while varying the period over which the trend in η and η_S^g are determined for the HR-CESM and LR-CESM and for (a & b): region 1 and (c & d): region 2. The minimum period over which the trends are determined is 25 years (same length as observations, Figure S5). The insets show the normalised SDSL trend where the period over which the trends are determined is set to 25 years, the dashed line indicates the normalised SDSL trend over the entire period (2000 – 2100).

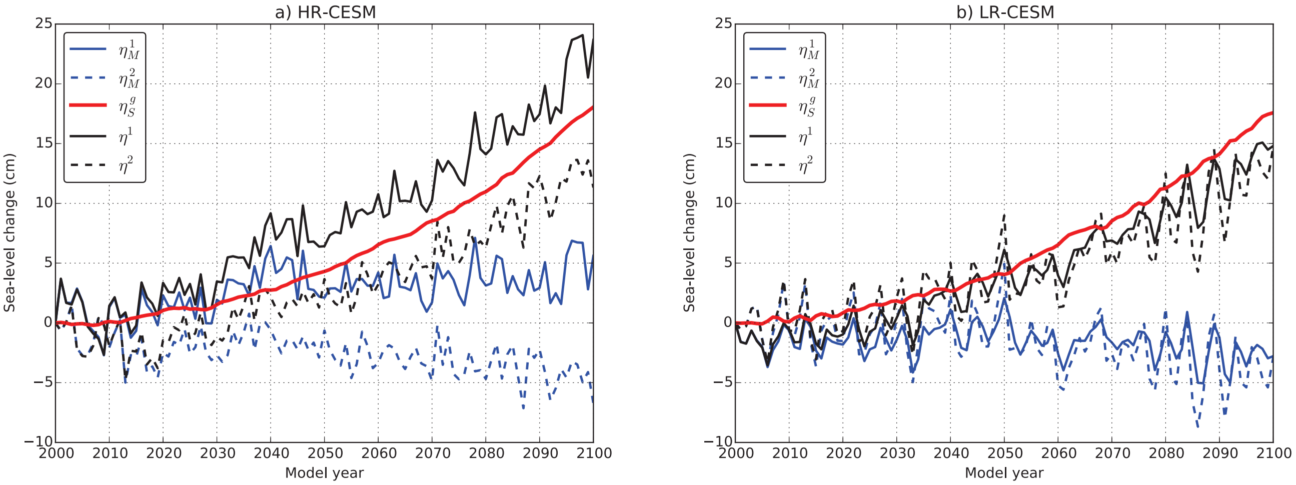

Figure S7. (a & b): Changes in the yearly-averaged DSL (η_M^x), the global-mean thermosteric sea-level (η_S^g) and SDSL (η^x), where x indicates the two different regions (cf. Figure 1) for the HR-CESM and LR-CESM. All time series are expressed as an anomaly with respect to model year 2000.

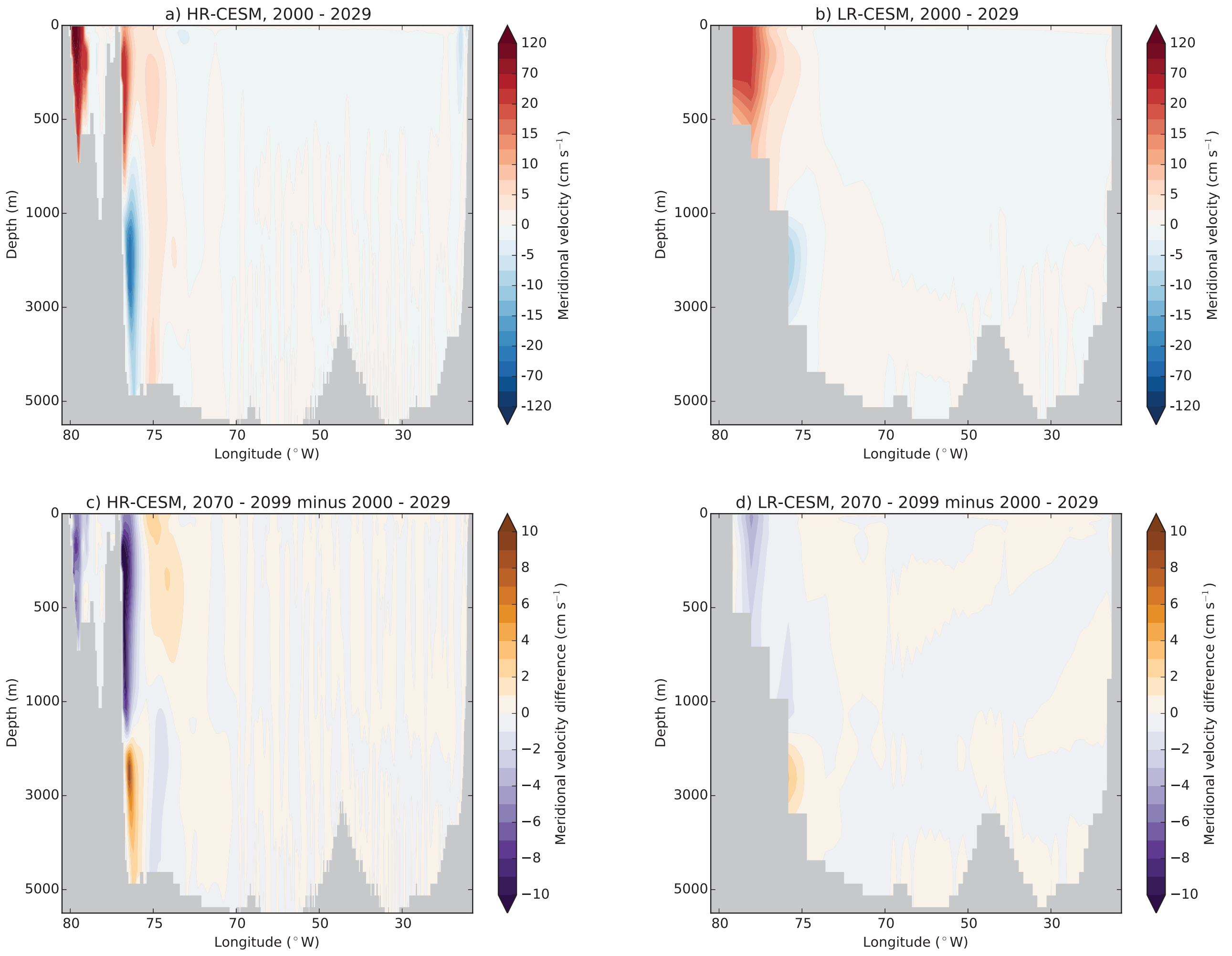

Figure S8. (a & b): Time mean over 2000 – 2029 of the meridional velocity along 26°N (i.e. AMOC transect) for the HR-CESM and LR-CESM. (c & d): Difference in the meridional velocity for the HR-CESM and LR-CESM, between the time mean over 2070 – 2099 and time-mean over 2000 – 2029.

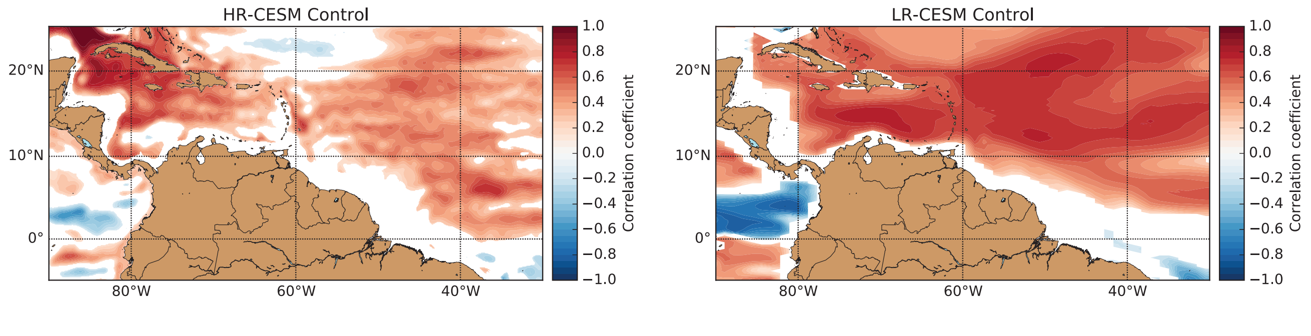

Figure S9. Correlation coefficient between the yearly-averaged η_M and BSF fields over the 101-year period for the HR-CESM Control and LR-CESM Control. Only significant (95%-confidence level) correlations are shown.

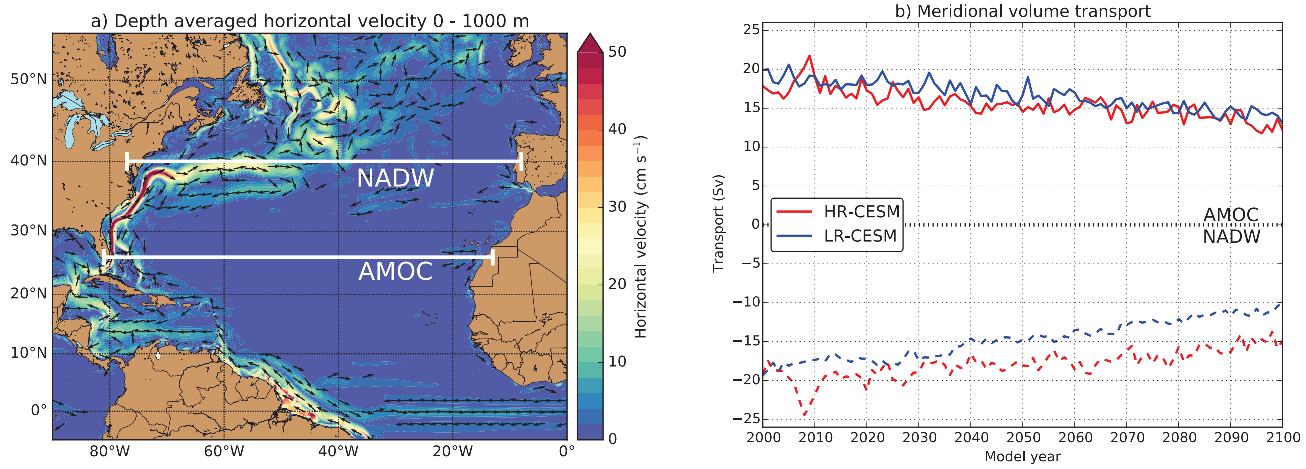

Figure S10. (a): Time-averaged (model years 200 – 300) horizontal velocity strength (colours) over the upper 1000 m of the HR-CESM Control simulation. The arrows indicate the direction of the flow (not to scale) and are only shown for horizontal velocities larger than 2 cm s^{-1}. The AMOC and NADW strength are determined along the white zonal sections, over the upper 1000 m (i.e. maximum in AMOC strength) and between 1500 – 4000 m (depth range is based on salinity and temperature properties of sea water), respectively. (b): Total meridional volume transport for the AMOC- and NADW strength. The trend in the AMOC is -5.0 $\pm$ 0.4 Sv per century (HR-CESM) and -5.4 $\pm$ 0.3 Sv per century (LR-CESM) and is for both significant (99%-confidence level). The trend in the NADW is 5.1 $\pm$ 0.4 Sv per century (HR-CESM) and 8.1 $\pm$ 0.2 Sv per century (LR-CESM) and is for both significant (99%-confidence level). Note that we reversed the vertical axis for the NADW strength in Figures 2c,d.

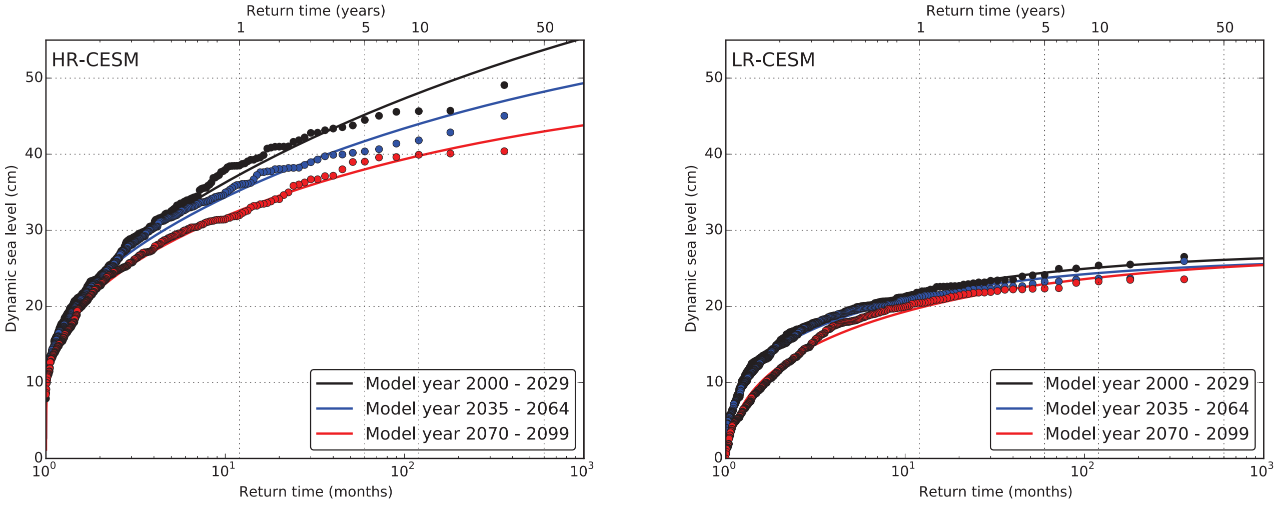

Figure S11. The GEV fit (curves) for the different periods of the η_M^{Max} time series (see Figure 3) for the HR-CESM and LR-CESM. The colour-coded dots are the η_M^{Max} time series on which the GEV is fitted.

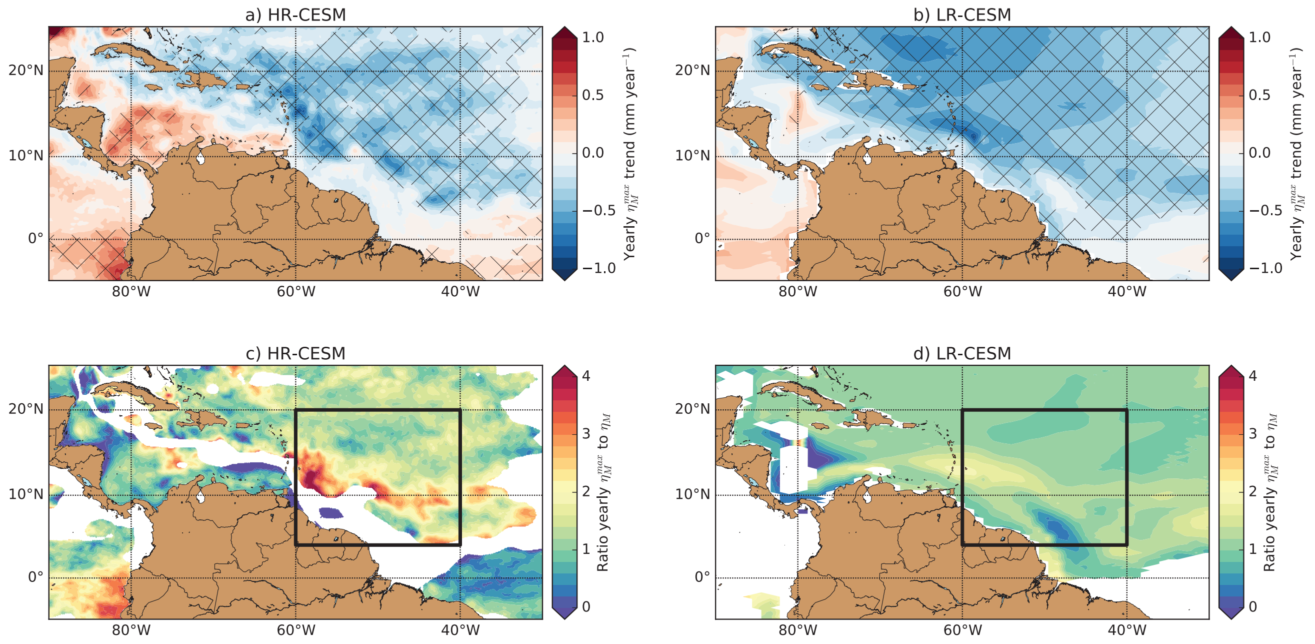

Figure S12. (a & b): The yearly η_M^{max} trend over the 101-year period for the HR-CESM and LR-CESM. The hatched regions indicate significant (95%-confidence level). (c & d): The ratio between the yearly η_M^{max} trend and the yearly-averaged η_M trend (cf. Figure S3). Regions which have a relatively small trend (± 0.1 mm year^{-1} or slower) in the yearly-averaged η_M trend are masked. The black outlined region is the NBC outflow region (cf. Figure 3).

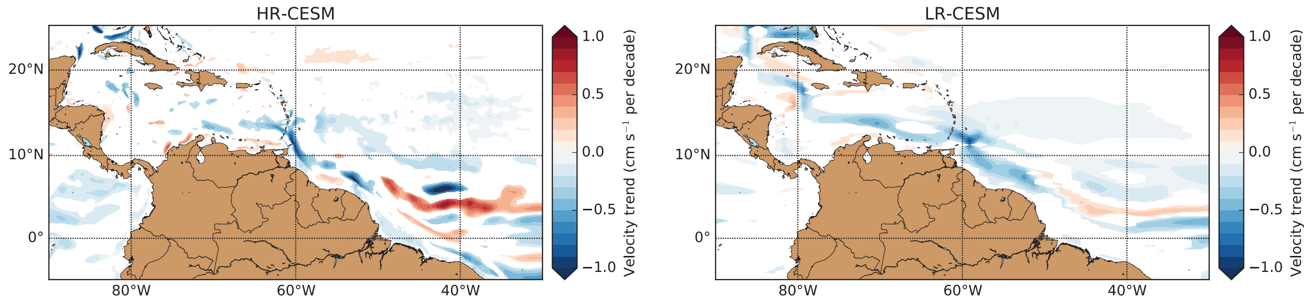

Figure S13. Upper 100 m horizontal velocity trend over the 101-year period for the HR-CESM and LR-CESM. Only significant (95%-confidence level) trends are shown.

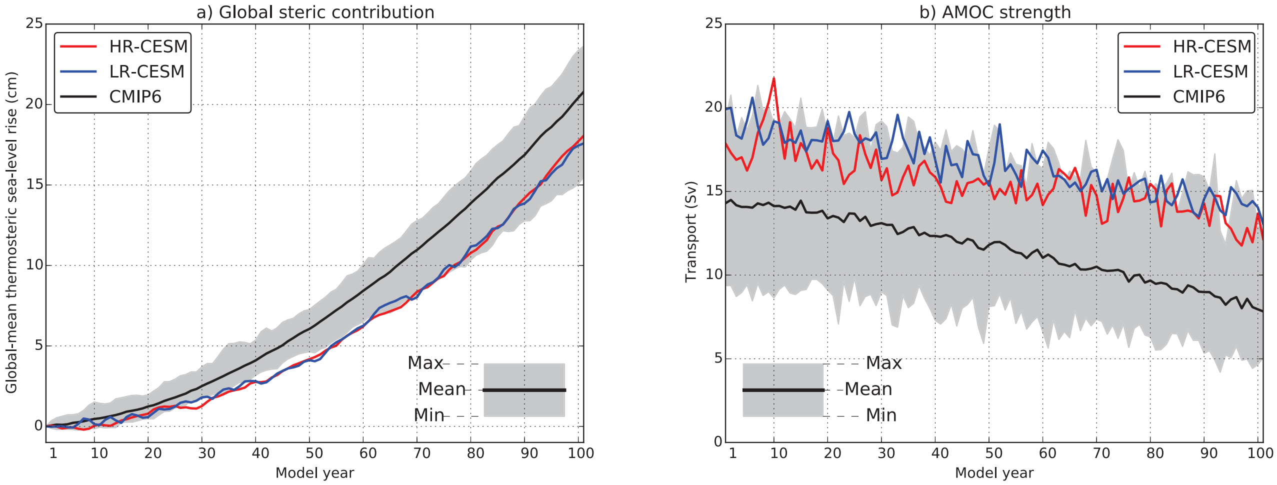

Figure S14. (a): Global-mean thermosteric sea-level rise for the HR-CESM, LR-CESM and the CMIP6 models. (b): AMOC strength at 26°N and 1000 m depth for the HR-CESM, LR-CESM and CMIP6 models. The black curve indicates the mean of the CMIP6 models, the shading indicates the maxima and minima of the CMIP6 models for each year.

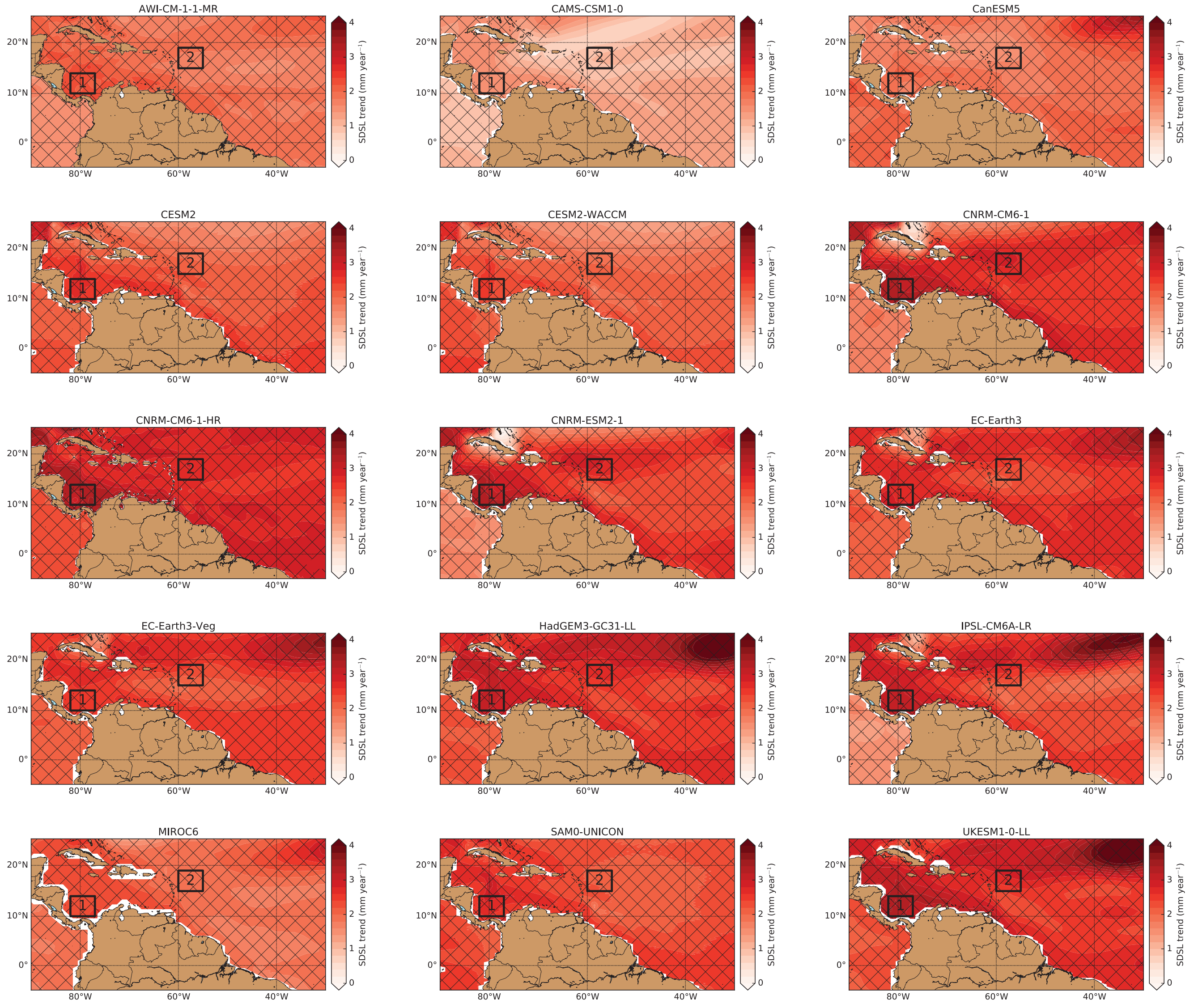

Figure S15. Local SDSL trend over the 101-year period for the CMIP6 models. The hatched regions indicate significant (95%-confidence level) trends. The SDSL trends are corrected for any η_S^g drift in the pre-industrial control simulations of the CMIP6 models.

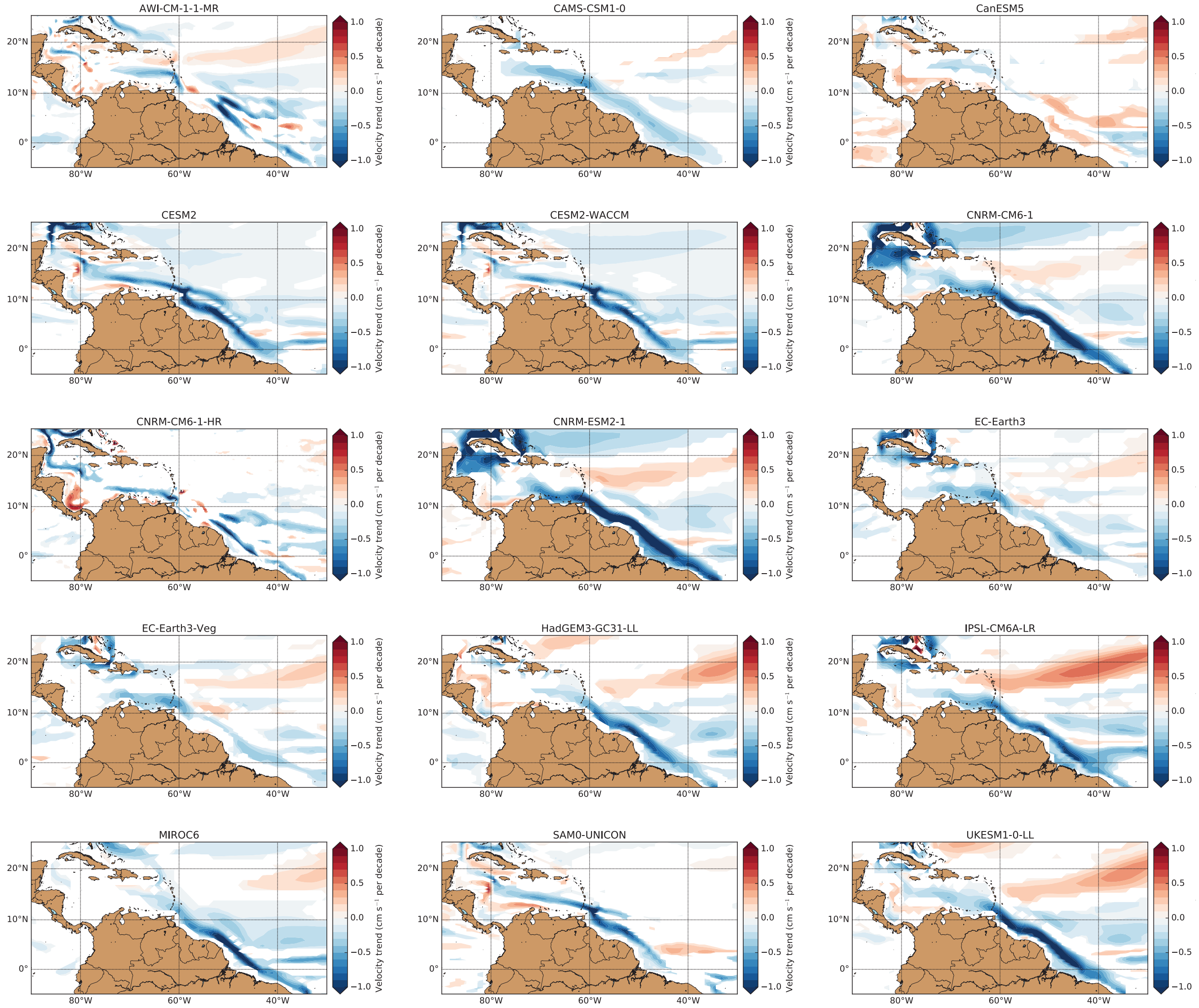

Figure S16. Upper 100 m horizontal surface velocity trend over the 101-year period for the CMIP6 models. Only significant (95%-confidence level) trends are shown.
